# Development of Symptomatic Bone Marrow Metastasis After Complete Response to Immunochemotherapy in Squamous Cell Lung Carcinoma

**DOI:** 10.1002/cnr2.70385

**Published:** 2025-11-28

**Authors:** Akari Momose, Tomonobu Koizumi, Risa Takei, Yutaro Waki, Hajime Midorikawa, Nami Kitagawa, Fumihiro Kawakami, Maki Ohya, Hideaki Hamano

**Affiliations:** ^1^ Department of Medicine Nagano Prefectural Kiso Hospital Nagano Japan; ^2^ Shinshu Cancer Center, Shinshu University Hospital Nagano Japan; ^3^ Department of Hematology and Medical Oncology Shinshu University School of Medicine Nagano Japan; ^4^ Department of Pathology Shinshu University School of Medicine Nagano Japan

**Keywords:** bone metastasis, immunochemotherapy, leukoerythroblastosis, non‐small cell lung cancer, thrombocytopenia

## Abstract

**Introduction:**

Symptomatic bone marrow metastasis in squamous cell lung cancer after achieving a complete response to immunochemotherapy has not been reported previously.

**Case Presentation:**

A 63‐year‐old Japanese man was referred to our hospital for further examination of an abnormal chest radiograph. Whole‐body computed tomography revealed left hilar swelling and osteolytic lesions. Bronchoscopic examination led to a diagnosis of squamous cell lung carcinoma with multiple bone metastases. He was treated with carboplatin/Nab‐paclitaxel and pembrolizumab, and achieved complete response as determined by positron emission tomography with fluorodeoxyglucose‐computed tomography. Despite pembrolizumab maintenance therapy for 1 year, thrombocytopenia, anemia, and leukoerythroblastosis occurred. Bone marrow biopsy revealed squamous cell carcinoma cells within fibrotic tissue, confirming the diagnosis of symptomatic bone marrow metastases. The patient died 1 month after this diagnosis.

**Conclusion:**

Recurrence presenting bone marrow metastasis should be considered in patients with squamous cell lung cancer, even achieving complete response to immunochemotherapy, particularly those with bone metastases.

## 1 Introduction

Symptomatic bone marrow metastasis can occur in nonhematological malignancies and is associated with poor prognosis [1, 2]. The most common primary nonhematological malignancies associated with bone marrow metastasis in adults are breast, stomach, prostate, and lung cancers [1, 2, 3, 4, 5, 6, 7, 8, 9]. Compared with small cell lung cancer [1, 2, 3, 10], symptomatic bone marrow metastasis is rare in non‐small cell lung cancer (NSCLC) [1, 2, 4, 5, 6, 7, 8, 9]. In addition, lung adenocarcinoma is the predominant histological type and bone marrow metastases from squamous cell lung cancer are further rare.

We present a case of squamous cell lung cancer that developed symptomatic bone marrow metastases, presenting with anemia, thrombocytopenia, and leukoerythroblastosis during pembrolizumab maintenance therapy. Although the patient initially had multiple bone metastases, he achieved a complete response (CR) to carboplatin/Nab‐paclitaxel and pembrolizumab.

## 2 Case Presentation

**FIGURE 1:**
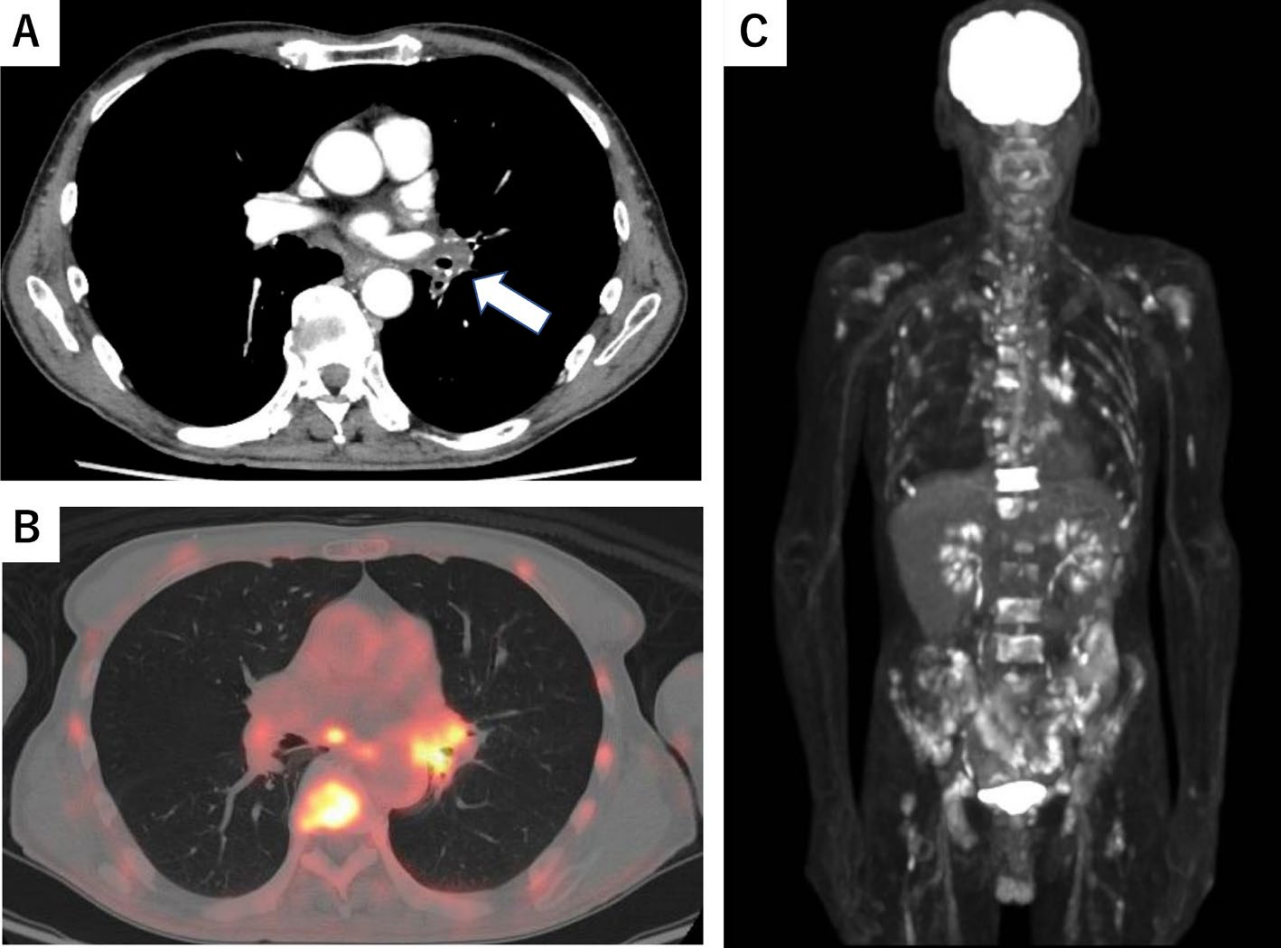
Radiographic findings at initial diagnosis and before immunochemotherapy. (A) Chest computed tomography revealed left main bronchial wall thickening and left hilar lymph node swelling. (B, C) Positron emission tomography with fluorodeoxyglucose‐computed tomography (FDG‐PET/CT) showed uptake in left hilar lymph node and multiple bone lesions, including the spine and the bilateral humerus and femur.

**FIGURE 2:**
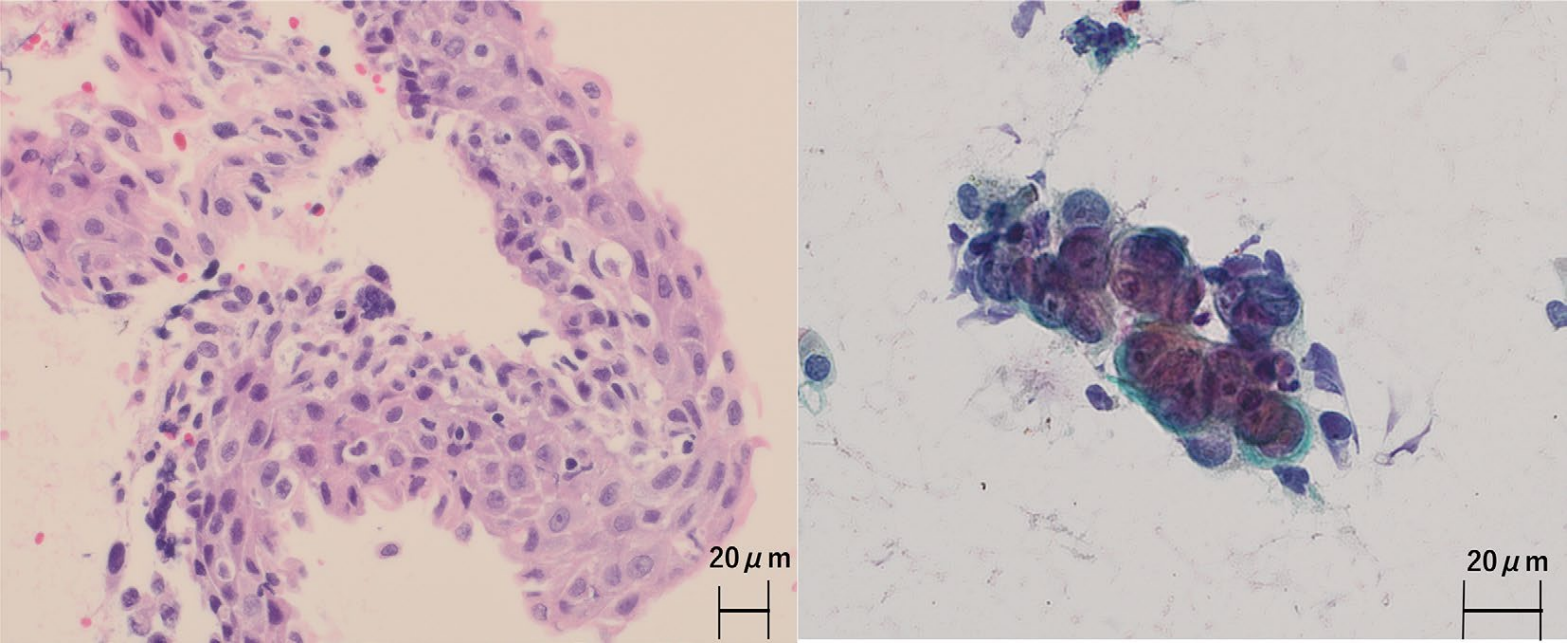
Histological findings of the lung specimen. (A) Hematoxylin and eosin staining. Histopathological findings at initial diagnosis by endobronchial ultrasound‐guided transbronchial needle biopsy specimens indicated the presence of squamous cell carcinoma. (×20). (B) Papanicolaou stain. Cytology in the specimen showed squamous cell carcinoma (×40).

**FIGURE 3:**
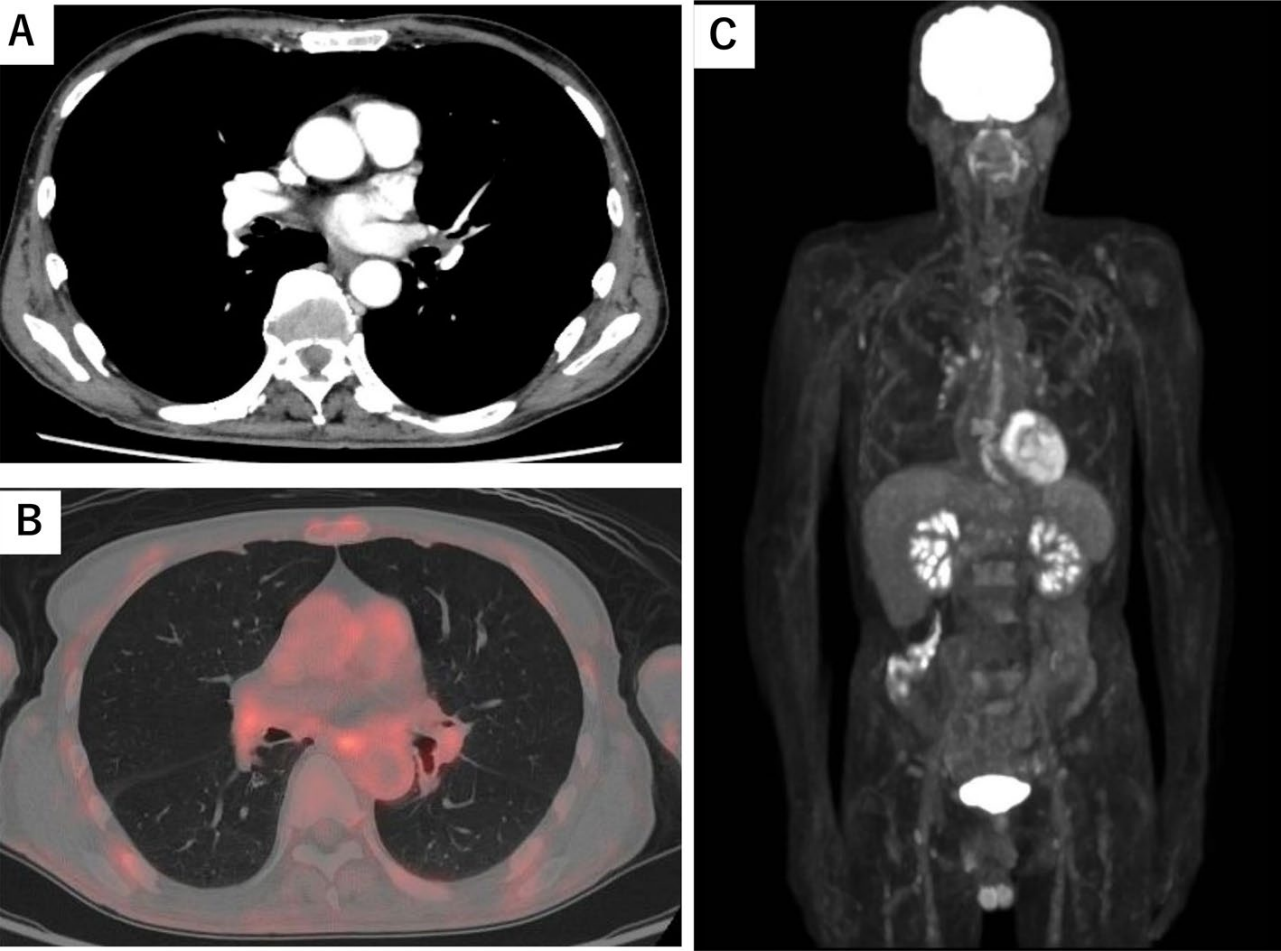
Radiographic findings after immunochemotherapy. (A) Chest computed tomography revealed resolution of left main bronchial wall thickening and left hilar lymph node swelling after immunochemotherapy. (B, C) Positron emission tomography with fluorodeoxyglucose‐computed tomography (FDG‐PET/CT) showed complete resolution of FDG uptake in all previously identified lesions after immunochemotherapy.

**FIGURE 4:**
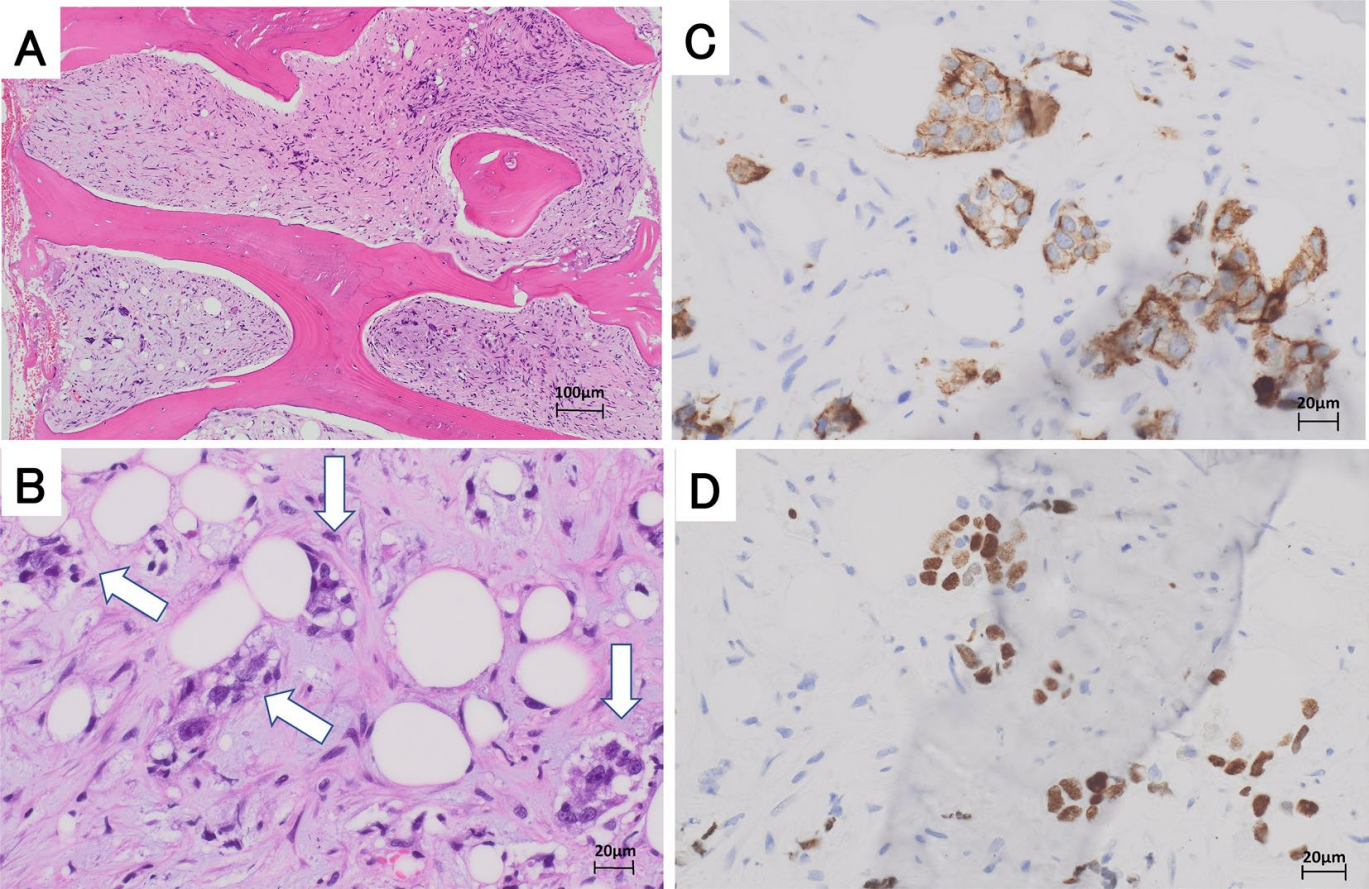
Histopathological findings of bone marrow specimen. (A, B) Hematoxylin and eosin staining in bone marrow biopsy specimen. Histopathological findings in bone marrow biopsy revealed squamous cell carcinoma cells around fibrotic tissue with myxomatous changes (A × 10, B × 40), arrows show the locations of tumor cells. (C) Immunohistochemical analysis for cytokeratin AE1/3. The tumor cells were positive for cytokeratin AE1/3 (×40). (D) Immunohistochemical analysis for anti‐ p40. The tumor cells were positive for p40 (×40).

A 63‐year‐old Japanese man who had a history of gastrectomy for gastric cancer 7 years previously had been followed up at Nagano Prefectural Kiso Hospital without recurrence. Whole‐body computed tomography (CT) performed in July 2023 revealed left main bronchial wall thickening and left hilar lymph node swelling (Figure 1A). Positron emission tomography with fluorodeoxyglucose‐computed tomography (FDG‐PET/CT) showed uptake in these areas (Figure 1B) and revealed multiple bone lesions, including the spine and the bilateral humerus and femur (Figure 1C). Endobronchial ultrasound‐guided transbronchial needle biopsy confirmed squamous cell carcinoma (Figure 2). The patient received 6 cycles of carboplatin (AUC 5), Nab‐paclitaxel (100 mg/m^2^), and pembrolizumab (400 mg), with no hematological toxicity or immune‐related adverse events. Chest CT showed resolution of left main bronchial thickening and disappearance of left hilar lymphadenopathy (Figure 3A). FDG‐PET/CT demonstrated complete resolution of FDG uptake in all previously identified lesions, indicating CR (Figure 3B,C). He continued pembrolizumab maintenance therapy for over 1 year, but was admitted to Nagano Prefectural Kiso Hospital because of general fatigue, appetite loss, and rapid progression of anemia and thrombocytopenia. The hemoglobin and platelet count declined from 11.7 g/dL (normal range: 13.7–16.6 g/dL) and 13.2 × 10^4^ μL (normal range 15.8–34.8 × 10^4^ μL) to 9.8 g/dL and 2.8 × 10^4^ μL for 1 month, respectively. Other laboratory findings were: white blood cells, 4000/μL (normal range 3300–8600/μL); lactate dehydrogenase, 560 IU/L (normal range: 124–222 IU/L); alkaline phosphatase, 183 IU/L (normal range 38–113 IU/L); and leukoerythroblastosis. Tumor markers were also elevated: SCC antigen, 4.8 ng/mL (normal range < 2.5 ng/mL) and CYFRA, 7.9 ng/mL (normal range < 3.5 ng/mL), both of which were within the normal limits at the time of the initial diagnosis of squamous cell carcinoma (1.2 and 2.3 ng/mL, respectively). Bone marrow aspiration yielded a dry tap, so bone marrow biopsy was performed. Histopathological examination revealed malignant cells surrounding fibrotic tissue with myxomatous changes (Figure 4A,B). Immunohistochemistry showed positivity for pan‐cytokeratin (AE1/AE3; Figure 4C) and p40 (Figure 4D), confirming bone marrow metastases from squamous cell lung carcinoma. The rate of programmed death‐1 (PD‐L1) expression was high in tumor cells (95%) using IHC 22C3 pharm Dx antibody. Few hematopoietic cell nests were observed in the bone marrow. The patient received multiple blood transfusions but died of aspiration pneumonia 1 month after admission.

## 3 Discussion

Previous clinical studies indicated that most bone marrow metastases occur in cases with adenocarcinoma histology, including cancers of the breast, stomach, prostate, and NSCLC [1, 2, 3, 4, 5, 6, 7, 8, 9]. Regarding squamous cell lung cancer, to our best knowledge, there were two case reports showing symptomatic bone marrow metastasis [11, 12]. Thus, bone marrow metastases originating from squamous cell lung cancer were extremely rare. In addition, it was also noteworthy in the present case that bone marrow metastasis eventually developed after a prolonged CR to immunochemotherapy. The clinical manifestation adds to the knowledge of a new clinical aspect of recurrence type during management in patients with lung cancer.

FDG‐PET/CT has high sensitivity for detecting bone marrow involvement in patients with malignancies [13]. In our case, FDG‐PET/CT 6 months after initiation of immunochemotherapy showed no abnormal FDG uptake in any systemic organs, including bone marrow. Therefore, the therapeutic response was assessed as CR to immunochemotherapy. Although the tumor cells demonstrated a high PD‐L1 expression (95%), pembrolizumab failed to control metastatic tumor cells in the bone marrow. Pathological examination revealed extensive fibrotic stromal changes surrounding scattered tumor cells, as reported previously in other cases of bone marrow metastasis [1, 2, 3, 4], which may have contributed to impaired microcirculation and led to inadequate drug delivery. Taken together, these observations suggest that residual tumor cells resistant to immunochemotherapy or undetectable by FDG‐PET/CT may have persisted during pembrolizumab maintenance therapy and subsequently contributed to the development of fibrotic stroma and tumor cell infiltration within the bone marrow space.

Several previous case series reported that most patients with bone marrow metastases also had concurrent bone metastases [1, 2, 3, 4, 5, 7]. This suggests that bone metastases could be a prevision for bone marrow involvement [3, 4, 7], because malignant cells in metastatic bone lesions could extravasate, disseminate, and infiltrate the bone marrow compartment.

In a meta‐analysis on molecular detection of tumor cells in bone marrow micrometastases in patients with early‐stage NSCLC after surgery, Deng et al. [14] reported no significant differences in the rate of bone marrow involvement between adenocarcinoma and squamous cell carcinoma. However, clinically documented symptomatic bone marrow metastasis has been reported predominantly in adenocarcinoma [1, 2, 3, 4, 5, 6, 7, 8, 9]. The mechanisms underlying symptomatic bone marrow metastasis development and progression are complex, and the reasons for the observed histological differences remain unclear.

The prognosis of NSCLC with symptomatic bone marrow metastasis is very poor [1, 4, 5, 6, 7, 8, 9], with reported survival ranging from only a few days or weeks to several months. Chemotherapy remains the only treatment option available. However, its use is dependent on the patient's physical condition and the extent of peripheral blood impairment. However, prolonged survival has been reported in patients with symptomatic bone marrow metastasis from *EGFR*‐ [9], *ERBB2*‐ mutated [15] or *ALK* fusion—positive [16] adenocarcinoma treated with tyrosine kinase inhibitors. These findings highlight the importance of comprehensive cancer multigene panel testing, even in cases of bone marrow metastasis in NSCLC. Unfortunately, our patient was not eligible for targeted molecular therapy and died 4 weeks after the diagnosis of bone marrow metastasis.

Anemia and thrombocytopenia are the most common clinical manifestations of bone marrow metastases. When these hematological abnormalities are unexplained, bone marrow metastasis should be considered in patients with NSCLC, particularly those with existing bone metastases.

## 4 Conclusion

We reported the first case of bone marrow metastasis originating from squamous cell lung cancer after a prolonged complete response to immunochemotherapy. This case highlights the importance of considering bone marrow involvement even in patients with NSCLC showing an excellent therapeutic response, particularly those with bone metastases.

## Author Contributions

A.M., T.K., R.T., Y.W., H.M., N.K., F.K., and H.H. contributed to treatment, data collection, and drafted the manuscript. M.O. contributed to the pathological diagnosis. T.K. is the corresponding author. All authors contributed to the article and approved the submitted version.

## Funding

The authors received no specific funding for this work.

## Ethics Statement

Ethics approval was not required for this study in accordance with local or national guidelines. Written informed consent was obtained from the patient's family for the publication of this case report and any accompanying images.

## Conflicts of Interest

The authors declare no conflicts of interest.

## Data Availability

The data that support the findings of this study are available from the corresponding author upon reasonable request.

## References

[1] H. S. Rani , M. Hui , P. L. Manasa , et al., “Bone Marrow Metastasis of Solid Tumors: A Study of 174 Cases Over 2 Decades From a Single Institution in India,” Indian Journal of Hematology and Blood Transfusion 38 (2022): 8–14. 35125707 10.1007/s12288-021-01418-9 PMC8804032

[2] M. H. Zhou , Z. H. Wang , H. W. Zhou , M. Liu , Y. J. Gu , and J. Z. Sun , “Clinical Outcome of 30 Patients With Bone Marrow Metastases,” Journal of Cancer Research and Therapeutics 14, no. Supplement (2018): S512–S515. 29970716 10.4103/0973-1482.172717

[3] A. Singh , S. Rawat , R. Kushwaha , et al., “Bone Marrow Metastasis in Nonhematological Malignancies: A Study From Tertiary Care Center,” Annals of African Medicine 23 (2024): 91–99. 38358178 10.4103/aam.aam_55_23 PMC10922175

[4] L. Xiao , S. Luxi , T. Ying , L. Yizhi , W. Lingyun , and P. Quan , “Diagnosis of Unknown Nonhematological Tumors by Bone Marrow Biopsy: A Retrospective Analysis of 10,112 Samples,” Journal of Cancer Research and Clinical Oncology 135 (2009): 687–693. 18956213 10.1007/s00432-008-0503-2 PMC12160163

[5] Y. S. Hung , W. C. Chou , T. D. Chen , et al., “Prognostic Factors in Adult Patients With Solid Cancers and Bone Marrow Metastases,” Asian Pacific Journal of Cancer Prevention 15 (2014): 61–67. 24528082 10.7314/apjcp.2014.15.1.61

[6] H. Yang , F. He , T. Yuan , W. Xu , and Z. Cao , “Clinical Features and Treatment of Bone Marrow Metastasis,” Oncology Letters 26 (2023): 332. 37415634 10.3892/ol.2023.13918 PMC10320432

[7] S. Gajendra and R. Sharma , “Cytomorphological Evaluation of Non‐Haematopoietic Malignancies Metastasizing to the Bone Marrow,” American Journal of Blood Research 13 (2023): 1–11. 36937461 PMC10017595

[8] R. Yang , L. Jia , and J. Cui , “Mechanism and Clinical Progression of Solid Tumors Bone Marrow Metastasis,” Frontiers in Pharmacology 15 (2024): 1390361. 38770000 10.3389/fphar.2024.1390361 PMC11102981

[9] D. Wang , Y. Luo , D. Shen , L. Yang , H. Y. Liu , and Y. Q. Che , “Clinical Features and Treatment of Patients With Lung Adenocarcinoma With Bone Marrow Metastasis,” Tumori Journal 105 (2019): 388–393. 30931812 10.1177/0300891619839864

[10] Y. Wang , J. Nong , B. Lu , et al., “Disseminated Tumor Cells in Bone Marrow as Predictive Classifiers for Small Cell Lung Cancer Patients,” Journal of the National Cancer Center 4 (2024): 335–345. 39735446 10.1016/j.jncc.2024.07.003 PMC11674436

[11] L. Lalwani , D. Chaundhry , S. Kumar , G. Arya , and P. K. Singh , “A Rare Site of Metastasis in Lung Cancer: Silent or Loquacious?,” Indian Journal of Case Reports 6 (2020): 317–320.

[12] R. Çiftçiler and C. Uğurluoğlu , “Squamous Cell Lung Cancer Presenting With Initial Rare Paraneoplastic Hematological Findings,” Acta Haematologica Oncologica Turcica 57 (2024): 121–122.

[13] K. M. Al‐Muqbel , “Bone Marrow Metastasis Is an Early Stage of Bone Metastasis in Breast Cancer Detected Clinically by F18‐FDG‐PET/CT Imaging,” BioMed Research International 2017 (2017): 9852632. 28884133 10.1155/2017/9852632 PMC5572575

[14] X. F. Deng , Q. X. Liu , D. Zhou , J. X. Min , and J. G. Dai , “Bone Marrow Micrometastasis Is Associated With Both Disease Recurrence and Poor Survival in Surgical Patients With Node‐Negative Non‐Small‐Cell Lung Cancer: A Meta‐Analysis,” Interactive Cardiovascular and Thoracic Surgery 21 (2015): 21–27. 25883247 10.1093/icvts/ivv082

[15] Y. Wu , J. Ni , X. Chang , X. Zhang , and L. Zhang , “Successful Treatment of Pyrotinib for Bone Marrow Metastasis Induced Pancytopenia in a Patient With Non‐Small‐Cell Lung Cancer and ERBB2 Mutation,” Thoracic Cancer 11 (2020): 2051–2055. 32458584 10.1111/1759-7714.13480 PMC7327666

[16] X. Li , X. Liu , F. Gao , and X. Yin , “Crizotinib Treatment in a Lung Adenocarcinoma Harboring ALK Fusion Gene With Bone Marrow Metastasis: Case Report and Literature Review,” Zhongguo Fei Ai Za Zhi 18 (2015): 85–88 (Chinese), PubMed Central. 25676401 10.3779/j.issn.1009-3419.2015.02.06 PMC5999841

